# Neurocysticercosis Diagnosed by *Taenia solium* PCR on Brain Biopsy

**DOI:** 10.1155/2020/8887647

**Published:** 2020-11-20

**Authors:** Sean Wei Xiang Ong, Jean-Marc Chavatte, Jonathan Wei Zhong Chia, Ramez Wadie Kirollos, Yih Yian Sitoh, Cristine Ding, Monica Chan

**Affiliations:** ^1^National Centre for Infectious Diseases, Singapore, Singapore; ^2^Department of Infectious Diseases, Tan Tock Seng Hospital, Singapore, Singapore; ^3^Department of Laboratory Medicine, Tan Tock Seng Hospital, Singapore, Singapore; ^4^Department of Neurosurgery, National Neuroscience Institute, Singapore, Singapore; ^5^Department of Neuroradiology, National Neuroscience Institute, Singapore, Singapore; ^6^Department of Pathology, Tan Tock Seng Hospital, Singapore, Singapore

## Abstract

Neurocysticercosis is a common cause for brain lesions and adult-onset epilepsy in endemic countries. However, diagnosis is challenging in the absence of typical radiologic or histopathologic features. In this case report, we present a case of a 35-year-old male with a new-onset seizure and a rim-enhancing temporal lobe lesion. Radiologic features were nonspecific, and brain biopsy was performed. Histologic features showed only nonspecific granulomatous inflammation, and the diagnosis of neurocysticercosis was confirmed only with polymerase chain reaction (PCR) testing on brain biopsy tissue demonstrating PCR products consistent with *Taenia solium*. This case highlights the diagnostic role of PCR in such clinical situations whereby the diagnosis is unclear after initial routine evaluation.

## 1. Introduction

Neurocysticercosis, caused by the tapeworm *Taenia solium,* has a worldwide distribution and is a common cause of adult-onset epilepsy in endemic countries [1]. The mainstay of diagnosis is via magnetic resonance imaging (MRI) of the brain demonstrating typical lesions of cysts with a mural nodule or scolex [1]. However, neuroimaging findings may be protean and not always pathognomonic. Serology is another diagnostic test that is commonly employed. However, various tests have varying performance in terms of sensitivity and specificity [2]. In this case report, we present a case of neurocysticercosis with nonspecific radiologic and histologic features, wherein diagnosis was confirmed with polymerase chain reaction (PCR) testing on brain biopsy tissue, highlighting the diagnostic role of PCR in such situations.

## 2. Case Report

The patient was a 35-year-old Indian male with no significant medical history. He was born in Tamil Nadu, India, and emigrated to Singapore six years prior to presentation. He returns to India annually to visit friends and relatives, both in small towns and villages in rural areas with farms housing farm animals. He drank well water and ate local food during these trips back to India. He had no known personal or contact history of tuberculosis.

He presented to medical care after a first-onset seizure which was witnessed by his wife. The seizure was described as jerking movements of all four limbs, associated with uprolling of eyeballs, and a transient loss of consciousness lasting ten minutes. There were no preictal symptoms. Postictally, he was drowsy for another ten minutes and was unable to recall the events upon regaining consciousness. Prior to this seizure, he was generally well and did not report any constitutional symptoms such as fever, night sweats, weight loss, or anorexia.

**Figure 1:**
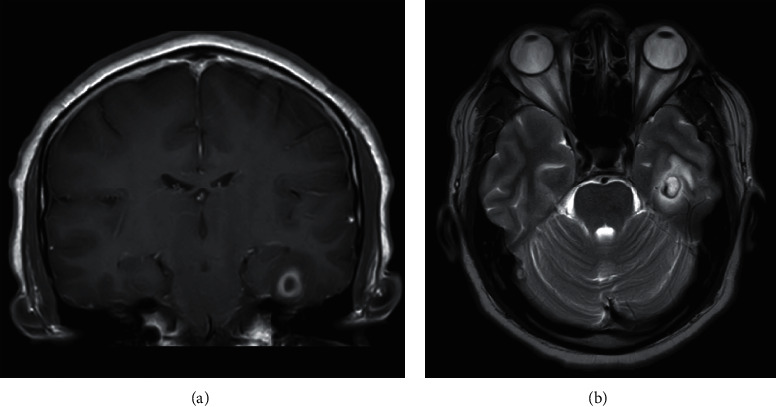
Magnetic resonance images of the left temporal lobe lesion. (a) Contrast-enhanced coronal T1-weighted image, showing rim-enhancing left temporal lobe lesion. (b) Axial T2-weighted image showing the same lesion with mild surrounding vasogenic edema.

On examination, he had a low-grade temperature of 37.8 degrees centigrade. Blood pressure, heart rate, and oxygen saturation were normal. Neurologic examination did not reveal any focal neurologic deficits, and there were no localising signs of infection. Laboratory investigations were significant for leukocytosis with a white blood cell count of 18.4 × 10^9^/L. There was no peripheral eosinophilia. Renal and liver function tests were normal, as were routine biochemistry and serum electrolytes. Noncontrasted computed tomography scan of the brain revealed an ill-defined eight-millimetre hypodense lesion with a hyperdense rim in the left basal temporal region, with mild surrounding vasogenic edema. MRI of the brain further delineated this as a solitary ring-enhancing lesion measuring 1.2 by 1 centimetre (Figure 1). Postcontrast images showed thick smooth peripheral enhancement, but no clearly-defined scolex or mural nodule consistent with a typical viable cyst of neurocysticercosis. Skull base artefacts limited assessment of the contents on diffusion-weighted images.

Human immunodeficiency virus serology was nonreactive. Two sets of blood cultures were negative, as was serum Cryptococcal antigen. Three sets of stool microscopy for ova, cyst, and parasites did not pick up any parasites. As there was no available serologic test for cysticercosis at our centre, a serum sample was sent to an overseas reference centre for serologic testing with an antibody-detecting ELISA-based assay.

After discussion with the managing neurosurgical team, the patient opted for brain biopsy for definitive diagnosis, in view of the broad range of differential diagnoses based on the neuroradiologic features. A left minicraniotomy was performed and identified a slightly hardened pale white to yellow lesion, with a milky white exudate upon breaching the lesion capsule. The entire cyst was excised and sent for histology and microbiologic evaluation.

**Figure 2:**
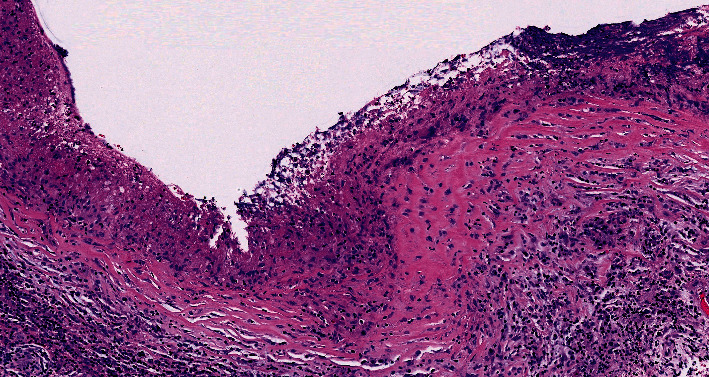
Histologic features of the resected left temporal lobe lesion. Fibrous cyst wall lined by aggregates of epithelioid histiocytes and multinucleated giant cells, along with necrosis and a mixed inflammatory infiltrate in the adjacent area (H&E, original magnification 200x).

Histology demonstrated granulomatous inflammation featuring aggregates of epithelioid histiocytes and multinucleated giant cells lining the fibrous cyst wall, accompanied by a mixed inflammatory infiltrate and necrosis. Features characteristic of a cysticercus were absent, and no viable larva was identified (Figure 2). No fungi or acid-fast bacilli were found on Gomori methenamine silver (GMS) and Ziehl–Neelsen histochemical stains, respectively, and no *Toxoplasma* organisms were identified on immunohistochemical staining.

Microbiologic testing did not identify causative organisms on Gram stain, aerobic and anaerobic culture, fungal smear, acid-fast smear, and GeneXpert MTB/RIF assay (Cepheid Inc, Sunnyvale, CA, USA).

In view of nonspecific histologic and radiologic features, no definite diagnosis was made at this point. As histology showed granulomatous inflammation, and tuberculosis is endemic in the region, empiric combination ant-tuberculous therapy and a tapering corticosteroid regimen were initiated after discussion and shared decision-making between the patient and medical team while awaiting further diagnostic tests.

**Figure 3:**
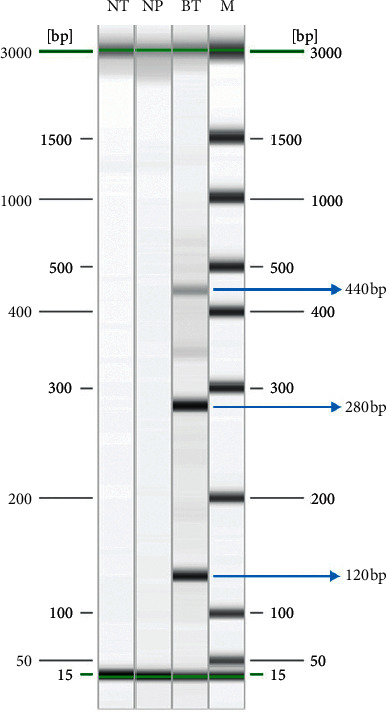
Amplicons from *Taenia solium*-specific PCR (pTsol9 repeat element) after capillary electrophoresis. Lanes NT, NP, and M represent no template, negative patient, DNA size marker. Lane BT represents brain tissue and shows multiple bands of ≈120 bp, 280 bp, and 440 bp, corresponding to the amplification of 1, 2, and 3 repeat unit(s) of the pTsol9 element, respectively.

While awaiting ELISA serologic assay results, which later returned positive for cysticercosis, residual brain tissue was sent to the National Public Health Laboratory, a tertiary reference laboratory, for further molecular diagnostic testing. 25 milligrams of brain tissue was minced on dry-ice, lyzed in ATL buffer with proteinase K, and DNA was extracted using QIAmp® DNA Mini Kit (Qiagen, Hilden, Germany) according to the manufacturer's instructions. A *Taenia solium*-specific PCR targeting the *pTsol9* repeat element based on a previous paper by Almeida and colleagues [3] and a pan-*Cestoda* PCR targeting a fragment of the mitochondrial ribosomal RNA (16S rRNA – 12S rRNA) genes based on a paper by Le and colleagues [4] were attempted. Capillary electrophoresis on QIAxcel® Advanced System (Qiagen, Hilden, Germany) equipped with DNA Fast Analysis Kit displayed multiple bands of expected sizes (120 bp, 280 bp, and 440 bp), corresponding to the amplification of 1, 2, and 3 repeat unit(s) of the *pTsol9* element, respectively (Figure 3) and a single band of 810 bp for the 16S rRNA-12S rRNA genes. Purification, bidirectional Sanger sequencing, alignment, and cross-checking of the sequences were performed as previously described [5] for the 16S rRNA-12S rRNA PCR products. A consensus fragment of the 16S rRNA-12S rRNA genes (808 bp) was obtained, compared by Basic Local Alignment Search Tool (BLAST) against GenBank (NCBI, Bethesda, MD, USA), showed 99.6% similarity (805/808) with sequence [AB086256], and thus ascertained that detected DNA was from *T. solium*. The sequence has been deposited in GenBank under the accession number (MT772182).

Upon molecular confirmation of neurocysticercosis, antituberculous therapy was stopped. The patient completed 14 days of oral albendazole together with a rapid taper of corticosteroids and has remained well since on subsequent follow-up.

## 3. Discussion

Diagnosis of neurocysticercosis remains challenging, and is often made presumptively on the basis of typical radiologic features, supported by consistent epidemiologic exposure and clinical presentation. The Infectious Disease Society of America guidelines for diagnosis and treatment of neurocysticercosis recommends neuroimaging and serologic testing as part of diagnostic evaluation, but recognizes the limitations of both these diagnostic modalities in terms of sensitivity and specificity [6]. Antigen assays on cerebrospinal fluid (CSF), serum, and urine are cited as an alternative, but are often not readily available.

However, the range of differential diagnoses for a solitary rim-enhancing brain lesion is wide, with both malignant and infective conditions [7]. Similarly, granulomatous inflammation on histology is nonspecific and has a wide range of possible infective and noninfective aetiologies [8]. Accurate diagnosis is vital for the administration of prompt, directed therapy, and to avoid delays in treatment for malignant or other infective aetiologies such as nocardiosis or tuberculosis [9]. In this case, definite diagnosis using PCR allowed for early tailoring of therapy, avoiding a prolonged course of empiric antituberculous drugs with its potential associated toxicities.

Molecular diagnostics have been utilized in numerous other parasitic diseases, improving diagnostic performance and turn-around time [10]. *T. solium*-specific PCR has similarly been used on CSF in the diagnosis of neurocysticercosis, with reported sensitivities of 72.2–95.9% and specificity of 100% [11–13]. However, to our knowledge, the use of direct PCR on brain tissue has not been reported in the literature, and we demonstrate that this is a useful diagnostic modality in similar clinical circumstances whereby the diagnosis is unclear after initial routine evaluation, and brain tissue is available for testing.

## Data Availability

The sequencing data used to support the findings of this study are available from the corresponding author upon request.
